# Ex Vivo Human Placental Transfer of Anti-Human Immunodeficiency Virus Compounds

**DOI:** 10.1155/S1064744997000537

**Published:** 1997

**Authors:** Roger E. Bawdon

**Affiliations:** Department of Obstetrics and Gynecology University of Texas Southwestern Medical Center 5323 Harry Hines Boulevard Dallas TX 75235-9032 USA

## Abstract

*Objective:* The transfer of anti-human immunodeficiency virus (HIV) drugs has been studied in the ex vivo human placental model. There is a paucity of information on the placental transfer of these drugs because of ethical considerations and the expense involved in the use of the non-human primate model.

*Methods:* The standardized ex vivo human placental model was used in these studies and the clearance index in relationship to antipyrine was used to determine the role of transfer of non-nucleosides, nucleosides, and a protease inhibitor. Several of the nucleosides and ritonavir were combined with zidovudine (AZT) to determine the effect of the combinations.

*Results:* All non-nucleosides, nucleosides, and the protease inhibitor were found to cross the human placenta by simple diffusion, although at variable rates. Ritonavir did not diffuse as rapidly as the nucleosides, but some diffusion was noted at peak concentrations.

*Conclusions:* Ex vivo perfusion studies agree with those determined in the non-human primate model and with data from existing clinical trials.


Infectious Diseases in Obstetrics and Gynecology 5:310–315 (1997)
© 1998 Wiley-Liss, Inc.

### Ex Vivo Human Placental Transfer of Anti-Human Immunodeficiency Virus Compounds

**Roger E. Bawdon***

*Department of Obstetrics and Gynecology, University of Texas Southwestern Medical Center, Dallas, TX*

### Abstract

*Objective:* The transfer of anti-human immunodeficiency virus (HIV) drugs has been studied in the ex vivo human placental model. There is a paucity of information on the placental transfer of these drugs because of ethical considerations and the expense involved in the use of the non-human primate model.

*Methods:* The standardized ex vivo human placental model was used in these studies and the clearance index in relationship to antipyrine was used to determine the role of transfer of non-nucleosides, nucleosides, and a protease inhibitor. Several of the nucleosides and ritonavir were combined with zidovudine (AZT) to determine the effect of the combinations.

*Results:* All non-nucleosides, nucleosides, and the protease inhibitor were found to cross the human placenta by simple diffusion, although at variable rates. Ritonavir did not diffuse as rapidly as the nucleosides, but some diffusion was noted at peak concentrations.

*Conclusions:* Ex vivo perfusion studies agree with those determined in the non-human primate model and with data from existing clinical trials. Infect. Dis. Obstet. Gynecol. 5:310–315, 1997.
© 1998 Wiley-Liss, Inc.

Key words perfusion; diffusion; nucleosides; protease inhibitors

In 1995, of the more than 500,000 cases of seropositive human immunodeficiency virus (HIV) in the United States, 14.5% or more than 72,000 women were HIV positive.[1] Also, in 1993, 6,530 HIV-infected women gave birth to an estimated 1,630 HIV-infected infants.[2] In addition, there were an estimated 12,240 HIV-infected children living in 1994. With these statistics, it is obvious that perinatal transmission of HIV is an important problem which needs to be addressed. With the completion of the Pediatric AIDS Clinical Trials Group Protocol 076, it was determined that the use of azidothymidine (AZT) reduced the transmission rate of HIV from mother to infant from approximately 24% to about 8%.[3] With this reduction in perinatal transmission, the use of other nucleoside and non-nucleoside inhibitors alone and in combination with protease inhibitors during pregnancy may become extremely important.

Because HIV mutates very readily and becomes resistant to single drug therapy, the use of combination therapy with two nucleoside inhibitors, and more aggressively two nucleoside inhibitors and a protease inhibitor, may be necessary for the prevention of perinatal transmission of the virus. The purpose of this review is to examine the published perfusion model data involving non-nucleoside and nucleoside inhibitors alone and in combination. Studies on the combination of a nucleoside inhibitor and a protease inhibitor also are included.

*Correspondence to: Dr. Roger E. Bawdon, Department of Obstetrics and Gynecology, University of Texas Southwestern Medical Center, 5323 Harry Hines Boulevard, Dallas, TX 75235-9032.

Received 3 June 1997

Accepted 8 October 1997

Review Article

### LITERATURE REVIEW

Studies on the pharmacology and teratology of the anti-HIV nucleosides in animal models and ex vivo systems are useful prior to the use of these inhibitors for treatment of HIV positive pregnant women. Among the nucleosides involved in teratology studies were 2′-3′-dideoxycytidine (ddC), 2′-3-dideoxyinosine, which is now didanosine (ddI), and AZT. In rat embryo culture studies, a minimum of 40% of embryos developed abnormality with AZT, while other drugs showed a higher interference with embryonic development. In experiments with pregnant rats treated with 200 mg/kg of each drug 3 times/day (600 mg/kg), there were no subsequent abnormal developments noted on day 10.[4,5] However, subsequent studies did identify pregnancy resorption at dose levels 15 times the normal human dose of 300 mg BID (2,250 mg/kg) under similar conditions. This is consistent with several studies showing the embryolethal effect of postimplantation in rats and preimplantation of mouse embryos.[4–8]

The current American College of Obstetricians and Gynecologists (ACOG) recommendation for the treatment of pregnant women is 100 mg of zidovudine (AZT) 5 times/day beyond 14 weeks of gestation and continued throughout pregnancy. Treatment during labor consists of intravenous zidovudine with a 2 mg/kg loading dose over 1 h followed by a continuous infusion of 1 mg/kg/h. Neonatal treatment consists of 2 mg/kg of zidovudine for the first 6 weeks of life beginning 8–12 h after birth.[9] With new guidelines about to be published, the above therapy is probably inadequate, as HIV drug resistance is known to develop on single drug therapy. The revisions include treatment with two nucleosides, and possibly two nucleosides and a protease inhibitor.

### Ex Vivo Perfusion Model

The model utilized in this study has been used successfully by other investigatiors to determine the transport of amino acids, lipids, electrolytes, metabolites, antimicrobial drugs, and anti-HIV compounds.[10,11] The criteria for the standardization of this model involve the use of standard media, monitoring fetal pressure, temperature, pH, and $O_2$ content, and the use of the reference compound antipyrine. The use of antipyrine, a freely diffusible small molecule, determines the maternal-fetal match in the isolated cotyledon as well as the basis for the clearance index (Ci), which is a percentage of the study compound based on the transfer of antipyrine. Thus the higher the Ci, the more readily the compound is diffusible and crosses the placenta. The formula used is as follows:

$$T_F = (C_{FV} - C_{FA})/(C_{MA} - C_{FA})$$

where $T_F$ = the transport fraction; C = concentration; M = maternal perfusate; F = fetal perfusate; A = artery; and V = vein.

In our study, the steady-state experiment in which the maternal and fetal circulation was not recirculated, the fetal artery contained none of the test substance, therefore:

$$T_F = C_{FV}/C_{MA}$$

and the transport fraction was used to derive the clearance (Cl) of the compound studied, where Q is the flow rate of the fetal circulation.

$$Cl = T_F \times Q.$$

Similar calculations were done with the test compound, in this case the anti-HIV drugs, so that the Ci is determined.

$$Ci = \frac{\text{clearance of test compound}}{\text{clearance of antipyrine}}.$$

In general, several concentrations of a test drug were used to coincide with the therapeutic trough and peak of the drug and a 10× concentration to determine saturation of the placental transport (diffusion) system. In addition, most concentrations were done in at least three different placentas.[12]

### HIV Replication and Inhibition

HIV is in the family Retroviridae, which encompasses a large number of infectious agents. One of the most important features of this family is its replication cycle. Retroviruses replicate from a single stranded genome of polyadenylated RNA. The virion also contains several enzymes important in its replication, which include reverse transcriptase, ribonuclease, and protease. The replication

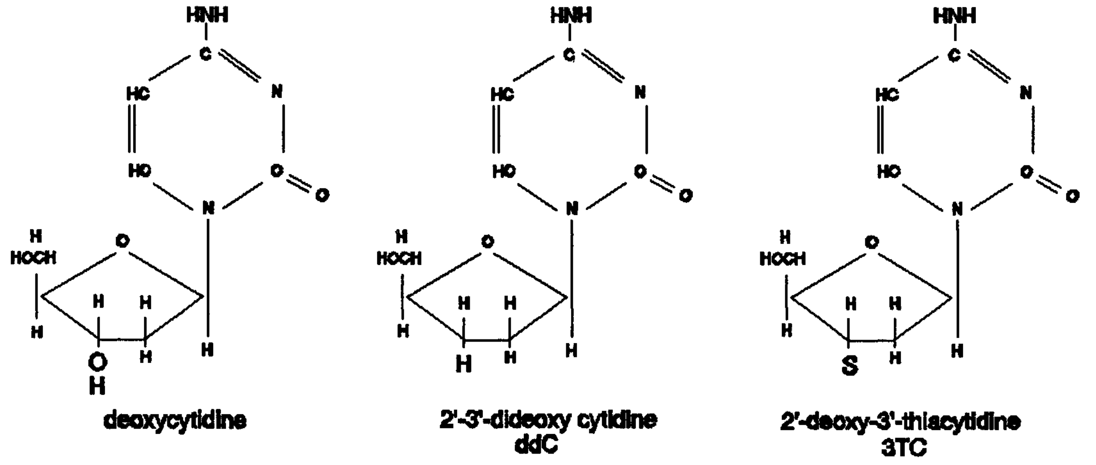

Fig. 1. Structural change in deoxycytidine to form ddC and 3TC, which causes viral RNA chain termination.

process involves cell entry, the coding of the viral RNA into double stranded DNA by reverse transcription, and ribonuclease. Once the process is completed, the viral DNA is integrated into the host DNA as a provirus where new viral RNA is formed and viral proteins are synthesized, leading to the completion and release of the virus.[13]

The focus of anti-HIV therapy has been in the prevention of transcription by the reverse transcriptase and in the inhibition of the protein cleavage at the termination of the formation of the virion; thus the terms "reverse transcriptase inhibitors" (both nucleoside and non-nucleoside) and "protease inhibitors." By altering the structure of natural nucleotides such as the removal of a free 3'OH group or the insertion of a sulfur atom in the ribose sugar of ddC, the compound may have an additional inhibitor effect. Intracellularly, 2'-dideoxy-3'-thiacytidine (3TC) is converted by cellular enzymes to the 5' triphosphate metabolite. By competing with the natural substrate 3TC 5' triphosphate and its incorporation into viral DNA, the analog (3TC) prevents formation of the 5'3' phosphodiester linkage essential for DNA chain elongation, therefore resulting in viral DNA termination. When these compounds are incorporated into growing chains, no further nucleosides can be added (chain termination). The alterations are shown in Figure 1.[13–15]. These compounds are inhibitory because their alterations cause changes in molecular configuration, which causes transcription termination.

Non-nucleoside inhibitors may inhibit reverse transcriptase by acting as the phosphate group on the sugar structure of the nucleotide, thus preventing incorporation into viral DNA.

Protease inhibitors include a wide variety of compounds which inhibit HIV protease (aspartic proteinase) activity. One such compound, ritonavir, has a molecular weight of 720 and binds competitively to the protease enzyme, to cause the production of immature virons. Mutations that occur in the viral genome may be related to resistance to protease inhibitors.

### Ex Vivo Human Placental Model and Nucleoside Inhibitors

Studies done with AZT used the isolated single cotyledon model with antipyrine as a freely diffusible marker.[16–18] Using these methods, it is possible for several laboratories to compare the rate of transport of various drugs. Several ex vivo human placental studies reported that the Ci of AZT was 0.24 ± 0.04 to 0.29 ± 0.06.[16–18] Although the placental perfusion model cannot be compared to the pregnant woman for pharmacokinetic parameters, unusual transfer or placental effects may be detected.

In vivo AZT pharmacokinetic studies in three pregnant HIV positive women were done at 19, 30, and 33 weeks with no unusual side effects. Amnionic fluid levels were high in the cord blood levels, suggesting fetal renal excretion and accumulation of the drug by the infant in the amnionic fluid.

In addition, cord blood levels were 113–127% higher than maternal levels.[19] These data confirm transplacental transport and fetal accumulation in both the in vivo and the ex vivo human placental

TABLE 1. Ex vivo placental transfer of anti-HIV nucleosides

| Trade name | Nucleoside inhibitor | Concentration (μg/ml)[a] | Ci[b] |
|---|---|---|---|
| Retrovir[22] | 3′-Azido-2′-3-dideoxyinosine (AZT) | 1 | 0.31 |
| Glaxo Wellcome (Research Triangle Park, NC) | AZT | 10 | 0.29 ± 0.04 |
| Videx | 2′-3′-Dideoxyinosine (ddI) | 1 | 0.14 ± 0.03 |
| Bristol-Myers Squibb (Syracuse, NY) | ddI | 10 | 0.15 ± 0.04 |
| Hivid | 2′-3′-Dideoxycytidine (ddC) | 1 | 0.22 ± 0.06 |
| Hoffman-La Roche (Nutley, NJ) | ddC | 10 | 0.23 ± 0.03 |
| Zerit | 2′-3′-Didehydro-3′-deoxythymidine (d4T) | 1 | 0.24 ± 0.07 |
| Bristol-Myers Squibb | d4T | 10 | 0.235 ± 0.045 |
| Epivir | (–) 2′-Dideoxy-3′-thiacytidine (3TC) | 1.4 | 0.23 ± 0.14 |
| Glaxo Wellcome | 3TC | 14.0 | 0.14 ± 0.06 |

[a]Approximate concentration.
[b]Standard deviation.

models. Other nucleoside compounds that are either available for treatment of HIV infections or are in various phases of clinical development and have been studied in the ex vivo human perfusion model include ddI, ddC, 2′-3′-didehydro-3′-deoxythymidine (d4T), and 3TC, of which the latter may only be used in combination with other nucleoside inhibitors including AZT.[20,21] The Ci of these compounds is depicted in Table 1.

Further studies, with these nucleoside inhibitors indicated there were no significant changes in the Ci when ddI, ddC, d4T, and 3TC were used in combination with therapeutic and 10x concentrations of AZT. When endogenous bases and the inhibitor dipyridamole were added to placental perfusion studies with these anti-HIV nucleoside inhibitors, there were no changes in the Ci, suggesting no transport system and no adverse effects of the combination. Thus, the compounds cross from the maternal to the fetal compartment by simple diffusion.[16–21]

In a recent open label pilot study (NUCB2018) in South Africa using 3TC alone and AZT along with 3TC in combination, 20 HIV pregnant women were given 8 mg/kg/day (300 mg BID) of 3TC or 4 mg/kg/day (150 mg/kg BID) 3TC plus 8 mg/kg/day of AZT at 38 weeks and continuing through 1 week postpartum. There was no viral RNA found in all 20 newborns at birth and 2 weeks postpartum. Maternal and fetal blood concentrations were similar to the levels used in the ex vivo human placental studies, and similarly, there were no changes in the blood concentrations when 3TC and AZT were used in combination. These data are promising, but further larger long-term studies are needed.[23]

### In Vivo Non-Human Primate Studies

Placental perfusion studies must be done in either non-human primates or in ex vivo perfusion models because anatomically no other animal has a discoidal placenta which has a villous fetomaternal interdigitation, a hemomonochorial barrier, and is multivillous.[24] When ddI was infused into the macaques at human doses along with antipyrine, a Ci of 0.09 ± 0.04 was determined, which was not different from studies in the ex vivo perfusion model (0.14 ± 0.05).[18,25] The in vivo studies also indicated that with constant infusion there was no accumulation of ddI in the fetus.

Stavudine (d4T) administered to pregnant pigtailed macaques (*Macaca nemestrina*) resulted in an antipyrine Ci of 0.23 ± 0.04, which was identical to the results reported in the ex vivo human placenta model.[20,26] Although the data in animal studies are limited, there is no conflict with the data from the ex vivo human placental model. The disadvantage of the ex vivo human placental model relates to the lack of actual pharmacokinetics of the drugs as the time to peak plasma concentration ($t_{1/2}$), peak plasma concentration ($C_{max}$), and bioavailability.

### Protease Inhibitors and Ex Vivo Perfusion Model

With the recent Food and Drug Administration (FDA) approval of four new protease inhibitors for the treatment of HIV-1 infections, these drugs and their approval represent a major advance in the management of HIV infection.[27] These new drugs include saquinavir mesylate (Invirase), ritonavir (Norvir), indinavir sulfate (Crixivan), and nelfinavir

(Virocept). However, these drugs are not without adverse reactions, as they have a wide variety of side effects and have complicated drug interactions with other medications used in the treatment of AIDS and other diseases of the adult patient. Because of these toxic properties in the use of these potent inhibitors alone and in combination with other inhibitors, there is a need for long-term studies in the in vivo non-human primate and short-term studies in the ex vivo human placental model. The toxicity and teratology of these drugs may prevent the use of protease inhibitors in the pregnant patient. To date there has been only one study done with ritonavir in the ex vivo human placental model.[28] In this study, the Ci and trough levels were below the sensitivity of detection by high pressure liquid chromatography. At therapeutic peak concentrations the Ci was 0.138 ± 0.04, which suggests there was little transport across fetal membranes at low levels, but the drug does cross at peak concentrations. However, there was little accumulation when the system was closed to allow for accumulation in the fetal circulation for 1 h. The addition of AZT had no effect on the transfer of ritonavir at either peak or trough concentrations.[28]

### Non-Nucleoside Reverse Transcriptase Inhibitors

The non-nucleoside reverse transcriptase inhibitor used in these studies was bisheteroypiperazine (U-87201-E).[29] The studies with U-87201-E suggest a rapid diffusion of the drug at therapeutic concentrations of 1.0 and 20 μg/ml with a Ci of 0.72 ± 0.17. This Ci is twice that of AZT, and 5 times that of ddI. Placental tissue concentrations were near those of the maternal blood concentration, suggesting saturation.[29]

### CONCLUSIONS

The ex vivo human placental model has provided basic information as to the transport of a wide variety of anti-HIV compounds. In application to clinical practice, the placental perfusion model has provided information on the use of multiple drugs without adverse reactions to the transfer of other anti-HIV compounds.[30] This has been substantiated with the protocol NUCB2018 with AZT and 3TC, in which perfusion experiments predicted no adverse reactions and demonstrated that the drugs were readily transported across the placental membranes. At this point, it may be concluded that all of the compounds thus far tested cross the placental barrier except ritonavir, which appears to be limited. Because there is a wide variation in transfer and these data agree with the non-human primate model, it is an important preliminary step to determine the transport and toxicity of these compounds prior to clinical trials with these drugs alone and in various combinations.

### DISCUSSION

Questions that need to be answered concerning the use of single or multiple anti-HIV drugs during pregnancy include the following[1]:

1. What are the long-term effects of these drugs on the mothers and fetuses of future pregnancies? *Since anti-HIV drug therapy for pregnant women is in its early stages, it is difficult to predict the effect on future pregnancies, but in all probability there will be no problems.*
2. Does the reduction in viral load to non-detectable levels in blood and tissue mean the virus has been totally eradicated? *Total elimination of the virus is not possible with existing treatment, as the virus is integrated into the human genome; only replication of the virus is prevented.*
3. Can these compounds be used in the first and second trimesters of pregnancy? *Probably not the first trimester, but definitely in the second trimester. A register for anti-HIV drugs is being established similar to the use of acyclovir when early treatment was administered prior to, at, or after conception.*
4. With the possibility of total elimination of HIV from blood and tissue by the use of combination (triple therapy), is pregnancy still an option? *These drugs do not destroy the virus, they just prevent viral replication.*
5. The issue of standard of care must be addressed; if AZT therapy alone is good enough in non-

[1]Even more aggressive anti-HIV therapy is being recommended in the *Federal Register* in the draft of the "Guidelines for the Use of Antiretroviral Agents in HIV-Infected Adults and Adolescents" and the U.S. Public Health Recommendations for Use of Antiretroviral Drugs During Pregnancy for Maternal Health and Reduction of Perinatal Transmission of Human Immunodeficiency Virus Type 1 in the United States; request for comment.

pregnant patients, why is it not good enough for pregnant women? *Because the virus mutates readily, single therapy is not adequate. The CDC and FDA will probably recommend triple therapy to prevent development of HIV resistance.*

### REFERENCES

1. Centers for Disease Control and Prevention: First 500,000 AIDS cases—United States. JAMA 247:952–955, 1995.
2. Davis SF, Byers RH, Lindegran ML, et al.: Prevalence and incidence of vertically acquired HIV infection in the United States. JAMA 247:952–955, 1995.
3. Wilfert CM: Beginning to make progress against HIV. N Engl J Med 335:1678–1680, 1996.
4. Klug S, Lewandowski C, Merker HJ, Stahlmann R, Wildi L, Neubert D: In vitro and in vivo studies on the prenatal toxicity of five virustatic nucleoside analogues in comparison to acyclovir. Arch Toxicol 65:283–291, 1991.
5. Sieh E, Coluzzi ML, Cusella De Angelis MG, et al.: The effects of AZT and ddI on pre- and postimplantation mammalian embryos: An in vivo and in vitro study. AIDS Res Hum Retrovirus 8:639–649, 1992.
6. Gogu SR, Beckman BS, Agrawal KC: Amelioration of zidovudine-induced fetal toxicity in pregnant mice. Antimicrob Agents Chemother 362:370–374, 1992.
7. Toltzis P, Mourton T, Magnuson T: Effect of zidovudine on preimplantation murine embryos. Antimicrob Agents Chemother 37:1610–1613, 1993.
8. Christmas JT, Little BB, Knoll KA, Bawdon RE, Gilstrap LC: Teratogenic and embryocidal effect of zidovudine (AZT) in Sprague-Dawley rats. Infect Dis Obstet Gynecol 2:223–227, 1995.
9. American College of Obstetricians and Gynecologists: Educ Bull 232:1–8, 1997.
10. Challier JC: Criteria for evaluating perfusion experiments and presentation of results. Contrib Gynecol Obstet 13:32–39, 1985.
11. Schneider H, Huck A: Dual in vitro perfusion of an isolated lake of human placentas: Method and instrumentation. 13:40–47, 1985.
12. Fortunato SJ, Bawdon RE, Maberry MM, Swan KF: Transfer of ceftizoxime surpasses that of cefoperazone by the isolated human placenta perfused in vitro. Obstet Gynecol 75:830–833, 1990.
13. Levy J (ed): The Viruses: The Retroviridae. Vol 1. New York: Plenum Press, p 190, 1992.
14. Rawn JD: Biochemistry. Burlington, NC: Neil Patterson Publishing, p 621, 1989.
15. Wainberg MA, Gu Z, Gao Q, Arts E, et al.: Clinical correlates and molecular basis of HIV drug resistance (review). J Acquired Immune Deficiency Syndrome Suppl 1:S36–46, 1993.
16. Liebes L, Mendoza S, Wilson D, Dancis J: Transfer of zidovudine (AZT) by human placenta. J Infect Dis 161: 203–207, 1990.
17. Schenker S, Johnson RF, King TS, Schenken RS, Henderson GI: Azidothymidine (zidovudine) transport by the human placenta. Am J Med Sci 229:16–20, 1990.
18. Bawdon RE, Sobhi S, Dax J: The transfer of anti-human immunodeficiency virus nucleoside compounds by the term human placenta. Am J Obstet Gynecol 167:1570–1574, 1992.
19. Watts DH, Brown ZA, Tartaglione T, Burchett SD, et al.: Pharmacokinetic disposition of zidovudine during pregnancy. J Infect Dis 163:226–232, 1991.
20. Bawdon RE, Kaul S, Sobhi S: The ex vivo human placental transfer of the anti-HIV nucleoside compound d4T. Gynecol Obstet Invest 38:1–4, 1994.
21. Bloom SL, Dias KM, Bawdon RE, Gilstrap LC III: The maternal-fetal transfer of lamivudine in the ex vivo human placenta. Infect Dis Obstet Gynecol (in press).
22. Physicians' Desk Reference, 1997.
23. Moodly J, Moodly D, Pellay K, et al.: Antiviral effect of lamivudine along and in combination with zidovudine in HIV infected women. Abstract, 4th Conference on Retrovirus and Opportunistic Infections, 1997.
24. Benirschke K, Kaufmann P: Placental types. In Benirschke K, Kaufman P (eds): Pathology of the Human Placenta. 2nd ed. New York: Springer-Verlag, 1990.
25. Pereira CM, Nosbisch C, Winter HR, et al.: Transplacental pharmacokinetics of dideoxyinosine in pigtailed macaques. Antimicrob Agents Chemother 38:781–786, 1994.
26. Odinecs A, Nosbisch C, Keller RD, et al.: In vivo maternal-fetal pharmacokinetics of stavudine (2′,3′ didehydro-3′-deoxythymidine) in pigtailed macaques (*Macaca nemestrina*). Antimicrob Agents Chemother 40:196–202, 1996.
27. Deeks SG, Smith M, Halodniy M, Kahn JO: HIV protease inhibitors. A review for clinicians. JAMA 277:145–153, 1997.
28. Casey B, Bawdon R: Placental transfer of ritonavir with zidovudine in the ex vivo placental perfusion model. Abstract, Society for Gynecologic Investigation, 1997.
29. Roberts S, Bawdon RE, Sobhi S, Dax J, Gilstrap L III: The maternal-fetal transfer of bisheteroypiperazine (U-87201-E) in the ex vivo human placenta. Am J Obstet Gynecol 172:88–91, 1995.
30. Carpenter CCJ, Fischl MA, Hammer SM, et al.: Antiretroviral therapy for HIV infections in 1997. Updated Recommendations of the International AIDS Society—USA Panel. JAMA 277:1962–1969, 1997.
